# Infection, cases due to SARS-CoV-2 in rural areas during early COVID-19 vaccination: findings from serosurvey study in a rural cohort of eastern India

**DOI:** 10.1017/S0950268822000346

**Published:** 2022-03-03

**Authors:** Pujarini Dash, Asit Mansingh, Soumya Ranjan Nayak, Debadutta Sahoo, Debdutta Bhattacharya, Srikanta Kanungo, Jaya Singh Kshatri, Bijaya Kumar Mishra, Matrujyoti Pattnaik, Debaprasad Parai, Hari Ram Choudhary, Swetalina Nayak, Khokan Rana, Alice Alice, Ajay Kumar Sahoo, Kanhu Charan Mohanty, Prasantajyoti Mohanty, Chinki Doley, Hitesh Jain, Dasharatha Majhi, Pooja Pattanayak, Santosh Behuria, Soumya Panda, Somnath Bhoi, Sanghamitra Pati, Subrata Kumar Palo

**Affiliations:** ICMR–Regional Medical Research Centre, Chandrasekharpur, Bhubaneswar 751023, Odisha, India

**Keywords:** Coronavirus, sero-survey, rural area, epidemiology

## Abstract

COVID-19 serosurvey provides a better estimation of people who have developed antibody against the infection. But limited information on such serosurveys in rural areas poses many hurdles to understand the epidemiology of the virus and to implement proper control strategies. This study was carried out in the rural catchment area of Model Rural Health Research Unit in Odisha, India during March–April 2021, the initial phase of COVID vaccination. A total of 60 village clusters from four study blocks were identified using probability proportionate to size sampling. From each cluster, 60 households and one eligible participant from each household (60 per cluster) were selected for the collection of blood sample and socio-demographic data. The presence of SARS-CoV-2 antibody was tested using the Elecsys Anti-SARS-CoV-2 immunoassay. The overall seroprevalence after adjusting for test performance was 54.21% with an infection to case ratio of 96.89 along with 4.25% partial and 6.79% full immunisation coverage. Highest seroprevalence was observed in the age group of 19–44 years and females had both higher seroprevalence as well as vaccine coverage. People of other backward caste also had higher seropositivity than other caste categories. The study emphasises on continuing surveillance for COVID-19 cases and prioritizing COVID-19 vaccination for susceptible groups for better disease management.

## Introduction

COVID-19 caused by SARS-CoV-2 has become the most difficult public health issue of the current time. Globally more than 246 million confirmed cases have been reported from 221 countries with around 5 million deaths [1, 2]. India alone contributes around 34 million cases and is only behind the US in the number of confirmed cases [2]. Covishield and Covaxin were approved by the Central Drug Standards Control Organization (CDSCO) in India for limited use in an emergency circumstance [3]. On 16 January 2021, India began immunizing against COVID-19 with the two vaccines for the targeted population; healthcare workers in health facilities and frontline workers working at community level [4]. The vaccination programme was implemented in a phased manner starting with healthcare workers and persons over 60 years of age, followed by those with comorbidities and finally is now open to all eligible adults. IgM antibody against COVID-19 is found for the initial 2 months of infection and IgG antibody starts to appear 2 weeks post-infection and persists for several months [5, 6]. According to the national serosurveys of India, despite the escalating effort to increase testing facilities, the infection to case ratio (ICR) was close to 30:1 in the first serosurvey (May–June 2020) and 80–130:1 in the second serosurvey (August–September 2020) indicating poor reliability of considering case number alone to measure the spread of SARS-CoV-2 in a region [7, 8]. ICR is the ratio of number of actual infections to number of reported cases caused by the pathogen that causes the disease. During the early phase of COVID pandemic, the difference between number of cases and undetected cases was not that noticeable, but in the long run, the number of undetected infections plays a major role in reducing the number of susceptible populations within a community and in turn affects the estimation of achieving the herd immunity threshold. In the third national serosurvey (December 2020–January 2021), a higher seroprevalence of SARS-CoV-2 infection was detected in urban regions compared to rural areas, indicating that a considerable number of people in rural areas were still susceptible to SARS-CoV-2 infection [9]. The seroprevalence varies greatly depending on geographical location and population density [10]. Model Rural Health Research Unit (MRHRU), Tigiria is a research unit of Indian Council of Medical Research–Regional Medical Research Centre (ICMR-RMRC), Bhubaneswar, situated in a rural area of Odisha, India. Knowing seroprevalence in this community will allow us to conduct a risk-benefit analysis of healthcare services in the catchment area of the unit and help to develop strategies for improving access to medical care in the rural community.

The current serological survey was undertaken in the rural cohort of the MRHRU, Tigiria (which includes four catchment blocks: Athagarh, Tigiria, Badamba and Narasinghpur) during March–April 2021 to estimate the seroprevalence of SARS-CoV-2 and determine the associated socio-demographic risk factors. This study also gives an initial snapshot of immunisation coverage in the rural context of Odisha, India.

## Methodology

The study was approved by the institutional human ethics committee, ICMR-Regional Medical Research Centre, Bhubaneswar (ICMR-RMRCB/AHIEC-2020). For the survey, a population-based cross-sectional study design was used which was based on the sampling protocol adopted by national and state serosurveys [9, 11–13]. The study population was randomly selected from the community members of the rural cohort of the MRHRU, Tigiria (blocks: Athagarh, Tigiria, Badamba and Narasinghpur) during March–April 2021.

### Sample size

The requisite sample size for our study was calculated for a finite population of 244 152 with a 95% CI using the OpenEpi sample size calculator. The estimated sample requirement was 3544 (rounded off to 3730) with the assumption of 26% seroprevalence (*p*) which was reported in the previous third-round national serosurvey study with an absolute error of 2.5%, design effect of 3 and a non-response rate of 5%.

Sample size *n* = [DEFF × *Np*[1–*p*]]/[*d*^2^/*Z*^2^_1−*α*/2_ × [*N*–1] + *p* × [1–*p*]]

### Sampling framework

A multi-stage random sampling method was used in the survey in which the clusters (villages) from each block were identified using probability proportionate to size sampling. From each cluster, 60 households were identified using a systematic random sampling method, and from each household, one individual was selected. For each study block, 15 cluster villages (a total of 60 clusters) were selected and 60 study participants from each cluster were selected using the Troldahl-Carter-Bryant Grid for data and blood sample collection [14]. Participants who were above 18 years of age, staying in that village at least for the last 3 months and provided written informed consent to participate were included, whereas pregnant women, cognitive impairment and bed-ridden patients were excluded.

### Data collection

Information on socio-demographic data, the status of comorbidity, COVID-19 immunisation status, travel history, history of COVID-19 infection, the practice of different COVID-19 protocols was collected through an electronic Open Data Kit (ODK) by trained field investigators. The questionnaire used for the survey was based on our previous published serosurveys [12, 13]. Also, secondary data on COVID-19 positive cases and vaccination coverage in the study area were collected from the health system during the survey.

### Sample collection

For serological analysis, 4 ml of blood sample was collected from each participant by trained phlebotomists using proper aseptic measures. Samples were transported to MRHRU, Tigiria for centrifugation, storage, further testing and analysis. The serological test was carried out at the COVID-19 laboratory, ICMR-RMRC, Bhubaneswar. All these procedures were done following appropriate aseptic measures with cold chain management (2–8 ^°^C).

### Laboratory procedures

Antibodies in serum samples against SARS-CoV-2 were detected in Roche Cobas E411 using the electro-chemiluminescence immunoassay-based technique which works on the double-antigen sandwich assay principle. The specificity of Roche e411 kit was reported as 99.81% (95% CI 99.65–99.91%) and the sensitivity was 100% (95% CI 88.1–100%) when tested after 14 days post SARS-CoV-2 confirmation as per the manufacturer. The presence of SARS-CoV-2 antibody was tested using the Elecsys Anti-SARS-CoV-2 immunoassay that follows *in vitro* qualitative detection of antibodies (including IgG) to SARS-CoV-2 in human serum. The immunoassay uses recombinant protein of nucleocapsid (N) antigen for the detection of antibodies against SARS-CoV-2. The value was expressed in Cut-off Index (CoI) and a value of <1.0 was considered non-reactive and COI ≥ 1.0 as reactive.

### Data analysis

Data were analysed using STATA. Univariate analysis was done for analysing the seroprevalence according to demographic characteristics. Seroprevalence and age–gender standardised seroprevalence were estimated as proportion with 95% CI according to socio-demographic variables. To determine the association between socio-demographic variables on seropositivity, bivariate logistic regression was used. For ICR, while the number of infections was determined by extrapolating the seroprevalence (SARS-CoV-2 IgG-positive) among the age- and gender-specific population in the study area, the number of COVID-19 cases was as reported by state health officials (data as of 15 days prior to serosurvey date) for the study area. ICR was calculated using the formula, ICR = estimated no. of infections (seroprevalence × population of the area)/reported cases (as reported in govt. database). The maps provided in the manuscript were generated using QGIS software (Open source) version 3.20.

## Result

**Fig. 1.**
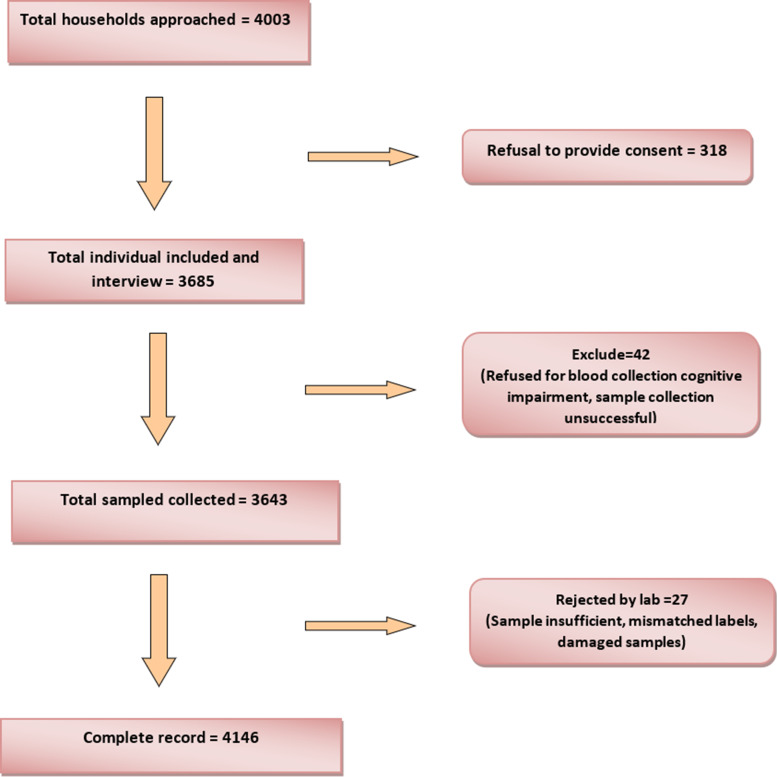
The process of recruiting study participants (*n* = 3643).

Under this study, we approached a total of 4003 participants and enrolled 3643 participants for data collection. After data cleaning and matching with the laboratory report, 3622 study participants were enrolled for final data analysis with 9% non-response rate. The flowchart about participant recruitment is given in Figure 1.

Though seropositivity among females (55.95%) was higher than males (52.74%), it was not statistically significant. Vaccination coverage among females was also higher (9.49%) as compared to males (4.12%) (Table 1). Seropositivity was seen highest among adult group (19–44 years; 55.91% (53.24–58.55%)) followed by the middle-aged group (45–59 years; 55.34% (52.41–58.24%)). The coverage of both the two doses of COVID-19 immunisation was highest among the adult group (10.05%) followed by middle aged (8.64%). Among participants aged 60 years and above, only 0.55% were vaccinated with both the doses of COVID-19 vaccine. In contrast, the coverage of a single dose of COVID-19 vaccine was highest (10.16%) among older age group and lowest (1.22%) among adults. Of note, seropositivity was found to be higher among unvaccinated individuals with COVID-19 history (82.60%) compared to vaccinated people having previous COVID-19 infection (76.66%) (Supplementary Table S2).

**Table 1.** Characteristics of the study population and their seroprevalence and vaccine coverage

Characteristic	Ab against SARS-CoV-2					Vaccination status		
	Population *N*	Positive (*n*)	Seroprevalence % (95% CI)	Seroprevalence % (95% CI)^a^	*P* value	Unimmunised	Partially immunised	Fully immunised
Gender								
Male	1820	960	52.74 (50.42–55.06)	52.74 (50.45–55.02)	0.054	1657 (91.04%)	88 (4.83%)	75 (4.12%)
Female	1802	1008	55.93 (53.60–58.24)	55.95 (53.57–58.31)		1565 (86.84%)	66 (3.66%)	171 (9.48%)
Age (years)								
Adult 19–44	1393	777	55.77 (53.12–58.40)	55.91 (53.24–58.55)	0.050	1236 (88.72%)	17 (1.22%)	140 (10.05%)
Middle aged 45–59	1157	642	55.48 (52.57–58.37)	55.34 (52.41–58.24)		1029 (88.93%)	28 (2.42%)	100 (8.64%)
Aged 60 and above	1072	549	51.21 (48.17–54.24)	51.02 (47.98–54.05)		957 (89.27%)	109 (10.16%)	6 (0.55%)
Ethnicity								
General	1108	581	52.43 (49.44–55.41)	52.35 (49.35–55.32)	**<0.01**	962 (86.82%)	64 (5.77%)	82 (7.40%)
OBC	1925	1100	57.14 (54.89–59.36)	57.11 (54.85–59.34)		1721 (89.40%)	79 (4.10%)	125 (6.49%)
SC	457	231	50.54 (45.86–55.22)	50.44 (45.74–55.13)		430 (94.09%)	9 (1.96%)	18 (3.93%)
ST	132	56	42.42 (33.87–51.32)	42.74 (34.14–51.68)		109 (82.57%)	2 (1.51%)	21 (15.90%)
Education								
No formal education	731	365	49.93 (46.24–53.61)	49.65 (45.94–53.36)	0.033	684 (93.57%)	46 (6.29%)	1 (0.13%)
Primary	1364	770	56.45 (53.77–59.10)	56.37 (53.68–59.03)		1274 (93.40%)	57 (4.17%)	33 (2.41%)
Secondary	988	532	53.84 (50.67–56.98)	53.81 (50.63–56.96)		864 (87.44%)	28 (2.83%)	96 (9.71%)
University	539	301	55.84 (51.53–60.08)	56.06 (51.78–60.28)		400 (74.21%)	23 (4.26%)	116 (21.52%)
Occupation								
Agriculture	677	373	55.09 (51.26–58.88)	55.02 (51.24–58.76)	**<**0.01	652 (96.30%)	24 (3.54%)	1 (0.14%)
Private sector	548	316	57.66 (53.40–61.84)	57.72 (53.52–61.84)		524 (95.62%)	20 (3.64%)	4 (0.72%)
Public sector	330	187	56.66 (51.12–62.08)	56.53 (50.98–61.96)		121 (36.66%)	28 (8.48%)	181 (54.84%)
Housewife	1415	805	56.89 (54.26–59.48)	56.88 (54.19–59.53)		1370 (96.81%)	41 (2.89%)	4 (0.28%)
Retired/unemployed	480	193	40.20 (35.78–44.74)	40.04 (35.65–44.54)		439 (91.45%)	39 (8.12%)	2 (0.41%)
Others	172	94	54.65 (46.89–62.24)	54.97 (47.19–62.57)		116 (67.44%)	2 (1.16%)	54 (31.39%)
Household size								
1–2	239	126	52.71 (46.18–59.18)	52.54 (45.96–59.05)	0.544	203 (84.93%)	22 (9.20%)	14 (5.85%)
3–5	2265	1220	53.86 (51.78–55.93)	53.79 (51.71–55.86)		2017 (89.05%)	71 (3.13%)	177 (7.81%)
>6	1118	622	55.63 (52.66–58.57)	55.60 (52.63–58.54)		1002 (89.62%)	61 (5.45%)	55 (4.91%)
COVID-tested positive								
No	3569	1926	53.96 (52.31–55.61)	53.90 (52.24–55.55)	**<**0.01	3199 (89.63%)	142 (3.97%)	228 (6.38%)
Yes	53	42	79.24 (65.89–89.15)	79.24 (65.89–89.15)		23 (43.39%)	12 (22.64%)	18 (33.96%)
Chronic diseases								
0	2288	1319	57.64 (55.59–59.68)	57.69 (55.63–59.73)	**<**0.01	2068 (90.38%)	51 (2.22%)	169 (7.38%)
1	719	381	52.99 (49.26–56.68)	52.78 (49.05–56.48)		626 (87.06%)	50 (6.95%)	43 (5.98%)
>1	615	268	43.57 (39.61–47.60)	43.32 (39.36–47.34)		528 (85.85%)	53 (8.61%)	34 (5.52%)

The values in bold are statistically significant (*P* < 0.05).

a Age and gender standardised.

Among the participants, 53 (1.46%) were already tested positive for COVID-19 of whom 42 (79.24%) were seropositive. Among 3569 (98.54%) who were either not tested or found negative in COVID-19 testing, 1926 (53.96%) were seropositive and the association was significant. A total of 2288 (63.17%) participants had no morbid condition and their age- and gender-standardised seroprevalence was 57.69%. In total, 1334 (36.83%) participants were suffering from one or more chronic diseases, of which 719 (19.85%) were having one chronic condition and 615 (16.98%) were having more than one chronic condition. The age- and gender-standardised seroprevalence among participants with single morbidity was 52.78% and among participants with multimorbidity was 43.32%. The distribution of seropositivity of participants according to socio-demographic characteristics is detailed in Table 1.

**Fig. 2.**
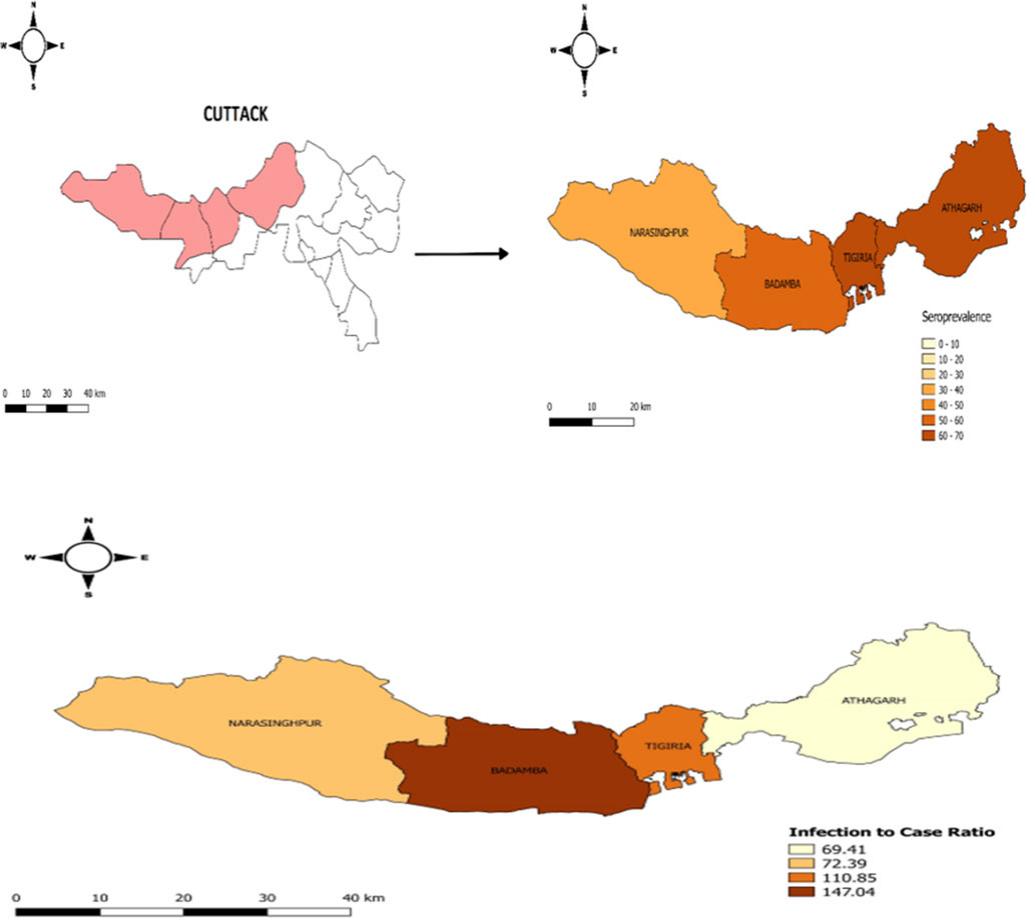
Heat map for blockwise prevalence and infection to case ratio.

The blockwise seroprevalence along with vaccination status is provided in Table 2. The overall unweighted seroprevalence was 54.33% (95% CI 52.69–55.96). The seroprevalence of the infection after adjusting for sensitivity and specificity of the test was 54.21% (95% CI 52.57–55.84) with the highest seroprevalence found in Tigiria block (63.19%) followed by Athagarh block (60.21%). Overall, 246 (6.79%) of study participants had completed their two doses of vaccination with the highest coverage from Narsinghpur block (12.34%). The overall ICR was 96.89 (95% CI 94.31–99.46). ICR was highest in the Narsinghpur block with 147.04 (95% CI 143.15–150.92) followed by Badamba 110.85 (95% CI 105.61–116.08). The blockwise seroprevalence and ICR is represented in Figure 2 and detail information regarding ICR calculation is provided in Supplementary Table S1.

**Table 2.** Blockwise seroprevalence and vaccination status

	Ab against SARS-CoV-2				Vaccination status			ICR (95% CI)	Total *N*
Blocks	Positive (*n*)	Seroprevalence % (95% CI)	Weighted seroprevalence^a^ % (95% CI)	Adjusted seroprevalence^b^ % (95% CI)	Unimmunised *n* (%)	Partially immunised *n* (%)	Fully immunised *n* (%)		
Athagarh	534	60.20 (56.89–63.44)	60.29 (56.98–63.53)	60.21 (56.90–63.45)	833 (93.91%)	5 (0.56%)	49 (5.52%)	69.41 (65.87–72.94)	887
Badamba	479	55.56 (52.17–58.91)	55.47 (52.08–58.83)	55.38 (51.99–58.73)	713 (82.71%)	103 (11.94%)	46 (5.33%)	110.85 (105.61–116.08)	862
Narsinghpur	395	39.97 (36.90–43.11)	39.79 (36.71–42.93)	39.67 (36.60–42.80)	832 (84.21%)	34 (3.44%)	122 (12.34%)	147.04 (143.15–150.92)	988
Tigiria	560	63.27 (60.00–66.46)	63.26 (59.98–66.45)	63.19 (59.92–66.37)	844 (95.36%)	12 (1.35%)	29 (3.27%)	72.39 (67.90–76.87)	885
**Total**	**1968**	**54.33 (52.69**–**55.96)**	**54.30 (52.65**–**55.93)**	**54.21 (52.57**–**55.84)**	**3222 (88.95%)**	**154 (4.25%)**	**246 (6.79%)**	**96.89 (94.31–99.46)**	**3622**

a Age and gender standardised.

b Adjusted for test performance.

Binary logistic regression model was run to study the effect of significant predictor variables on SARS-CoV-2 antibody development among study participants (Table 3). The ethnicity of the study subjects showed a significant higher seropositivity among the OBC group (OR 1.17, 95% CI 1.01–1.36). Household size of 8 or more had a higher odds of acquiring SARS-CoV-2-specific antibodies compared to those with household size 1–4 (OR 1.24, 95% CI 0.97–1.58) though the difference was not significant. COVID-19-positive patients had more than three times higher odds of developing the SARS-CoV-2 antibody compared to those who are either not tested or tested negative. Compared to people having chronic illnesses, individuals without any chronic illness were more likely to be seropositive (OR 1.31, 95% CI 1.12–1.51).

**Table 3.** Bivariate logistic regression model of COVID-19 seropositivity with socio-demographic characteristics

Characteristic	Sample (*n*)	Unadjusted odds ratio	Adjusted odds ratio
Gender			
Male	1820 (960)	Ref	Ref
Female	1802 (1008)	1.13 (0.99−1.29)	1.06 (0.82–1.37)
Age (years)			
19–44	1393 (777)	1.20 (1.02–1.41)	0.87 (0.71–1.06)
45–59	1157 (642)	1.18 (1.01–1.40)	0.96 (0.79–1.16)
60 and above	1072 (549)	Ref	Ref
Ethnicity			
General	1108 (581)	Ref	Ref
OBC	1925 (1100)	1.20 (1.04–1.40)	1.17 (1.01–1.36)
SC	457 (231)	0.93 (0.74–1.15)	0.98 (0.79–1.23)
ST	132 (56)	0.66 (0.46–0.96)	0.73 (0.50–1.07)
Education			
No formal education	731 (365)	Ref	Ref
Primary	1364 (770)	1.30 (1.08–1.55)	1.25 (1.03–1.51)
Secondary	988 (532)	1.17 (0.96–1.41)	1.08 (0.87–1.35)
University	539 (301)	1.26 (1.01–1.58)	1.21 (0.91–1.61)
Occupation			
Agriculture	677 (373)	Ref	Ref
Public sector	548 (316)	1.11 (0.88–1.39)	1.15 (0.90–1.47)
Private sector	330 (187)	1.06 (0.81–1.38)	0.99 (0.69–1.41)
Housewife	1415 (805)	1.07 (0.89–1.29)	1.08 (0.79–1.46)
Retired/unemployed	480 (193)	0.54 (0.43–0.69)	0.61 (0.47–0.78)
Others	172 (94)	0.98 (0.70–1.37)	0.99 (0.66–1.46)
Household size			
1–4	1751 (958)	Ref	Ref
4–8	1536 (812)	0.93 (0.81–1.06)	0.95 (0.82–1.10)
>8	335 (198)	1.18 (0.94–1.51)	1.24 (0.97–1.58)
COVID-tested positive			
Not tested or negative	3569 (1926)	Ref	Ref
Yes	53 (42)	3.25 (1.67–6.34)	3.40 (1.71–6.74)
Vaccination status			
No	3222 (1745)	Ref	Ref
Yes	400 (223)	1.06 (0.86–1.31)	1.04 (0.79–1.36)
Chronic conditions			
No	2232 (1286)	1.41 (1.23–1.61)	1.31 (1.12–1.51)
Yes	1390 (682)	Ref	Ref

The distribution of COVID-19 seroprevalence and ICR according to study blocks is depicted through Heat maps generated for different blocks under Figure 2.

## Discussion

Present COVID-19 serosurvey was carried out among 3622 participants aged 18 years and above during March–April 2021 in four catchment rural block areas of MRHRU, Tigiria, Cuttack. The mean age of study participants was 49.18 (±15.27) years. Among the participants, 50.24% were male, 54.33% were tested COVID-19-positive and 36.83% had one or more chronic illnesses. This survey showed that 54.21% of people had developed antibodies against SARS-CoV-2 by March–April 2021 in rural areas. The three rounds of national serosurveys had shown the rural seroprevalence of COVID-19 as 0.52% (May–June 2020), 5.2% (August and September 2020) and 21.4% (December 2020–January 2021). According to the national COVID-19 serosurvey, the seroprevalence in rural areas increased by about 2.5-fold from second to third round [8, 9]. A study in the urban slums of Bangalore city had found the COVID-19 seroprevalence of 57% during September 2020 [15]. Our findings indicate that by April 2021, the rural areas were not far behind from the COVID-19 infection transmission. Factors like migration of people from outside to their respective villages during the lockdown and more relaxed behaviour and practice among rural people towards adhering to COVID-19 protocols greatly attributed to this spread. According to Malani *et al*., in Bihar, a large share of workers returning from different parts of country were tested COVID-19 RT-PCR-positive [16].

Till the time of this serosurvey (March–April 2021), COVID-19 vaccination was initiated for frontline workers but not for all age groups, indicating that they must have been exposed to infection. Compared to overall seroprevalence (26%) in the third round of national serosurvey of India (December 2020–January 2021), seroprevalence of 54.21% in rural areas (March–April 2021) shows a significant rise [9]. This could be due to the fast spread of the infection and initiation of the COVID-19 vaccination. Though there are limited prior studies revealing any caste-wise variation in seroprevalence, our finding suggests that OBC category people have higher seroconversion compared to other castes. According to a study by Mondal and Karmakar (2021), SC category people are most socially vulnerable group in this COVID pandemic followed by OBC and forward caste people indicating safer status of forward caste where as both SC and OBC are lagging behind reflecting the impact of social inequalities [17]. With limited information to establish this plausibility, more research studies could help us to investigate it further. Among different occupation groups, participants working in private organisations or having business-related occupation were more likely for seropositivity compared to other occupation groups like housewives, agriculture workers and employees. This could be due to their work-related higher possibilities of exposure. Serosurveys in the capital city of the state, Odisha (same state in which the present study has been carried out), showed people doing public service (first round survey), people working in manufacturing (second round survey) and homemakers (third round survey) had highest seroprevalence (12). However, another serosurvey study carried out in a rural region of India showed no association between occupation and seropositivity (10).

The seroprevalence was higher among adult age group (19–44 years; 55.91%) compared to older people (60 years and above; 51.02%). Kshatri *et al*. (2021) had also found highest seroprevalence in 30–39 years age group in their community serosurvey in Bhubaneswar during September 2020 [12]. Similar prevalence pattern across the age groups was noticed in the third national serosurvey, which showed a higher seropositivity among 45–60 years age group compared to 60 years and above age group; however, the seroprevalence in the 18–44 years age group was lower compared to elder group [9]. In contrast to our observation, a study from a rural district of south India found no association of seropositivity with age [15]. According to a hospital-based study from Srinagar, people aged 30–69 years had a higher odds ratio of seropositivity than the younger population [18]. No significant gender difference pertaining to seropositivity was observed in our study and this is in line with the findings from many other studies [18–20]. In contrast to this, the national serosurvey reported a higher seroprevalence among male [21]. Age and gender influence the mobility, though it varies according to socio-cultural conditions. So, the probability for exposure can be attributed to the practice of mobility rather than age and gender. Comorbidity is another important factor that is associated with the severity of COVID-19 illness. A study on data published during January–April 2020 showed that, COVID-19 patients with comorbidities are at higher risk of having more severe disease [22]. Hypertension, chronic obstructive pulmonary disease, obesity, cancer, chronic liver and renal disease have been found to be associated with higher severity and greater probability of mortality in COVID-19 patients [23]. Though our study did not focus on the clinical severity, our study showed a contrasting finding indicating a negative association between comorbidity and having greater chance of COVID-19 infection. The presence of one or more chronic conditions affects the social behaviour of the person which ultimately might have impact on chances of getting COVID infection. A study among US adults reported that, people having health conditions such as cardiovascular, respiratory or immune-related diseases were more likely to work from home, avoid crowd and wear face masks to avoid further health complications during COVID pandemic period [24]. Staying back home, having less interaction with other people and extra safety measures might be helping these people in escaping COVID-19 infection to some extent.

A higher ICR (96.89; 95% CI 94.31–99.46) in our study explains the importance of serosurveys in understanding the COVID-19 situation better and in formulating appropriate strategies for effective pandemic management. According to the third round of the national serosurvey, the ICR was 26.7 (CI 25.4–28.0). This clearly implies the gravity of the COVID-19 infection situation, especially in rural areas. Factors such as low awareness, expenditure for travel, out-of-pocket expenses for availing healthcare, less accessibility to laboratory testing facility, fear of social isolation if tested positive contribute to the low seeking for testing and less reporting attribute to this [10, 15, 22, 25, 26]. This suggests prioritizing for the testing facility, more so in rural areas, in order to diagnose and manage the cases at an early stage. More similar studies at regular intervals are needed to understand the status of infection transmission better.

### Limitations

Our serosurvey was limited to only four rural blocks of Cuttack district, Odisha. Children below 18 years of age were not included in this study. Though the presence of chronic illness was studied, details about the chronic conditions were not considered. Considering the geographic limitation and rural context, the findings of this study need to be interpreted accordingly.

## Conclusion

COVID-19 seroprevalence of 54.21% along with ICR of 96.89 in rural areas till April 2021 imply the increased spread of the infection also in rural areas. However, there is a gap in detection and reporting of COVID-19 cases from these areas. In rural areas, challenges such as access to healthcare facilities, poor healthcare seeking and financial constraints for availing health services hinder them from health services including COVID-19-related services. Efforts have been undertaken by the department of health and family welfare with priority in tracing, testing and treating the cases through established COVID Care Centres (CCC). However, there is still a gap in early case detection, especially in rural areas. Many socio-cultural factors also attribute to these gaps and challenges. Appropriate context-specific strategies focusing on rural areas need to be undertaken in order to control and prevent the ongoing pandemic. COVID-related services such as COVID-19 testing, tracing, management, immunisation and health awareness among community people would be helpful to limit the disease transmission and contain the pandemic.

## Data Availability

The datasets for this study can be available from the corresponding author on reasonable request. Restrictions apply to the availability of individual-level data, in the interests of study participant confidentiality.

## Supplementary material

For supplementary material accompanying this paper visit https://doi.org/10.1017/S0950268822000346. click here to view supplementary material
