# Supplementary material for: Infection, cases due to SARS-CoV-2 in rural areas during early COVID-19 vaccination: findings from serosurvey study in a rural cohort of eastern India
Source: Epidemiol Infect. 2022 Mar 3;150:e58. doi: 10.1017/S0950268822000346 (PMC8937583; doi:10.1017/S0950268822000346)
Supplement: Supplementary file 1 [file S0950268822000346sup001.doc]

**Supplementary table-1: Detail information of calculation of Infection to case ratio (ICR = estimated no. infections / reported cases)**

| **Block** | **No. of reported Covid-19 cases** | **Estimated number of infections** | **Infection to case ratio (95% CI)** |
| --- | --- | --- | --- |
| Athagarh | 769 | 53379 | 69.41 (65.87 – 72.94) |
| Badamba | 566 | 83225 | 147.04 (143.15 – 150.92) |
| Narasinghpur | 539 | 39020 | 72.39 (67.90 – 76.87) |
| Tigiria | 426 | 47224 | 110.85 (105.61 – 116.08) |
| Total | 2300 | 222849 | 96.89 (94.31 – 99.46) |

**Supplementary table-2: Differential analysis of COVID-19 seropositivity with/without vaccination history**

| Groups | Seropositivity | |  |  |
| --- | --- | --- | --- | --- |
|  | Yes | No | Chi-sq value | p-value |
| Vaccinated history + COVID-19 history | 23 | 7 | 12.2 | <0.01 |
| Unvaccinated + COVID-19 history | 19 | 4 |
| Vaccinated without COVID-19 history | 200 | 170 |
